# Smoking fewer than 20 cigarettes per day and remaining abstinent for more than 12 hours reduces carboxyhemoglobin levels in packed red blood cells for transfusion

**DOI:** 10.1371/journal.pone.0204102

**Published:** 2018-09-26

**Authors:** Renata E. Boehm, Bruno D. Arbo, Denise Leal, Alana W. Hansen, Rianne R. Pulcinelli, Flávia V. Thiesen, Almeri M. Balsan, Tor G. H. Onsten, Rosane Gomez

**Affiliations:** 1 Postgraduate Program in Health Sciences, Universidade Federal de Ciências da Saúde de Porto Alegre (UFCSPA), Porto Alegre, Brazil; 2 Hemoterapy Center, Hospital de Clínicas de Porto Alegre (HCPA), Porto Alegre, Brazil; 3 Postgraduate Program in Pharmacology and Therapeutics, Universidade Federal do Rio Grande do Sul (UFRGS), Porto Alegre, Brazil; 4 School of Pharmacy, Pontifícia Universidade Católica do Rio Grande do Sul (PUCRS), Porto Alegre, Brazil; University of Athens, GREECE

## Abstract

**Background:**

The prevalence of smokers among blood donors and the effect of smoking on the quality of donated blood have not been extensively explored. In the present study, we determined the prevalence of smoker donors in a large blood bank in Southern Brazil and evaluated the quality of packed red blood cells (RBCs) from these donors through recommended quality control tests and measurement of carboxyhemoglobin (COHb) levels. We then assessed the influence of smoking habits and abstinence before donation on these parameters.

**Material and methods:**

An observational study was conducted to determine the prevalence of smoking donors, while a prospective cohort study compared conventional hematological and serological parameters and COHb levels at 0, 15, and 30 days after donation in RBCs donated by smokers (N = 31) and nonsmokers (N = 31) and their association with smoking habits and abstinence before donation.

**Results:**

Of 14,428 blood donations received in 1 year, 5.9% were provided by smokers. Storage over time slightly altered some quality parameters, such as hematocrit, hemoglobin, hemolysis, and COHb levels, in RBC packs. COHb levels were higher in RBC packs from smokers (8%) than from non-smokers (2%), and increased as a function of the number of cigarettes smoked daily and time elapsed since the last cigarette smoked before donation. Lower levels were found in RBC packs from donors who smoked fewer than 20 cigarettes per day or remained abstinent for more than 12h before giving blood.

**Conclusion:**

Although cigarette smoke had no significant effect on blood quality parameters such as hematocrit, hemoglobin, or hemolysis, it quadrupled COHb levels in packed RBCs. Abstinence from smoking for more than 12h or smoking fewer than 20 cigarettes daily helped decrease COHb levels.

**Implications:**

Given the increasing prevalence of tobacco use worldwide, we suggest blood banks recommend 12h of tobacco abstinence before donation and analyze COHb levels in donated blood as an approach to reduce risk for high-risk recipients.

## Introduction

Smoking is a major public health problem, and is considered the leading cause of preventable death worldwide [1]. About 21% of the world population smokes tobacco, and approximately 6 million people die each year of causes related to tobacco-smoke exposure, which represents one death every 6 seconds [1].

The particulate (tar) and vapor phases of tobacco smoke contain more than 4,700 substances [2]. Vapor-phase smoke carries products such as carbon monoxide, ammonia, ketones, formaldehyde, acetaldehyde, and acrolein [3], while particulate matter carries nicotine, heavy metals such as nickel, arsenic, cadmium, and lead, and other substances such as benzopyrenes [4]. Most of these products are toxic, promoting tissue damage secondary to oxidative stress and inflammation [2,4,5]. Carbon monoxide (CO), particularly, decreases the oxygen (O_2_)-transporting capacity of blood because of its much higher hemoglobin affinity (200 times that of O_2_) [6]. Carboxyhemoglobin (COHb), the byproduct of this reaction, is unable to carry O_2_, reducing its availability to tissues and moving the O_2_ dissociation curve to the right, which causes chemical asphyxia and hypoxia [6]. Although the deleterious effects of CO on the quality of blood for transfusion are known, few studies have evaluated the effect of donor smoking on the quality of packed red blood cells (RBCs) [7–9].

Blood transfusion is the oldest and most common therapeutic modality used to treat and prevent inadequate O_2_ release to tissues. As are other therapeutic modalities, it is associated with both acute and delayed adverse effects [10–12]. The major complications of blood transfusion are mostly related to hemolytic reactions [13], transfusion-related acute lung injury (TRALI) [14], and infections [12]; multiple organ failure, renal dysfunction, and mortality can occur [10]. To reduce these risks, blood donors are rigorously screened and selected following national and international rules for eligibility, defined in accordance with criteria for past medical history and physical examination, as well as hematological and serological testing [15,16]. Individuals who have used alcohol or marijuana 12h before donation, those who have used cocaine (including crack cocaine) in the preceding 12 months, chronic alcoholics, and all intravenous drug users are generally barred from donating blood [15,16]. However, there are no restrictions on smoker donors; the only advice given to this population is to abstain from smoking within 2h of donation, to prevent donor side effects such as dizziness and nausea [15,16]. The prevalence of smokers among blood donors and the effect of cigarette smoke exposure on the quality of donated blood have not been extensively explored [17–19]. Within this context, we aimed to determine the prevalence of smoking donors at the Blood Bank of Hospital de Clínicas de Porto Alegre (HCPA), a large tertiary referral center in Southern Brazil, and evaluate the quality of packed RBCs from these donors, assessing not only legally required blood-quality parameters but also COHb levels.

## Methods

This study was entered in the Plataforma Brasil registry (CAAE # 14002313.1.0000.5345) and approved by the HCPA Research Ethics Committee.

### Study design

An observational study was conducted from October 2013 to September 2014 to determine the prevalence of smoking donors at the HCPA Blood Bank in Porto Alegre, state of Rio Grande do Sul, Brazil. For this purpose, a question about smoking habits was included in the health history questionnaire administered to all potential donors.

In a convenience sampling strategy, donors of both sexes, aged 18–40 years, who confirmed a smoking habit, were invited to participate. All were informed about the aims and benefits of this study and signed an informed consent form before enrollment. Subsequently, they answered questions about the brand and number of cigarettes smoked per day, time since the last cigarette smoked before blood donation, lifestyle, and socioeconomic information. Sex- and age-matched non-smoker donors were invited to compose the control group. Blood donors who reported exposure to polluted environments, including tobacco smoke (second-hand smokers) or occupational exposure to burning wood/coal, automobiles, heavy machinery, or toxic gases, were excluded.

RBC samples were collected from packs donated by the smoker and non-smoker samples, and the remaining blood (~200 mL) was stored for transfusion purposes. The number of RBC packs to be monitored over the duration of the study was calculated (WinPepi^®^, 11.4, USA) assuming a mean (SD) 2% difference in blood COHb concentration between smokers and non-smokers, admitting a minimum difference of 0.25% in non-smokers, a statistical power of 90%, and an alpha error of 5%. The resulting sample size was calculated as 31 RBC packs per group [20].

### Sample collection and storage

Whole blood (±450mL) was collected in triple polyvinyl chloride bags (JP Farma^®^, Ribeirão Preto, Brazil) containing citrate phosphate dextrose adenine (CPDA-1) anticoagulant solution, centrifuged at 24 °C and 2,535 rpm for 5 min (KR 4i, Thermo Scientific^®^, USA), and its components separated by an automatic extractor (Giotto, Delcon^®^, Italy). All precautions to avoid red cell contamination were taken as per standard protocols. RBC packs did not receive any treatment (such as irradiation or leukoreduction). A 60-mL sample was collected from each RBC pack through a sterile connection and transferred to a paediatric transfusion pack with the same characteristics to those of the original pack. Samples were labeled and stored in the same refrigerator and at the same temperature (+2 to +6 °C) as the original packs. Analyses were carried out at three time points—zero (the day of donation) and every 15 days thereafter—to monitor changes in COHb levels and blood quality over time.

Recommended quality control tests were performed as per Brazilian legislation and relevant guidelines [15,16]. At each time point, 10-mL samples were taken from the pediatric transfusion pack by a single puncture, keeping the system closed. After each collection until the last time point, the RBC pack was returned to the refrigerator, thus reproducing standard blood storage.

### Quality of blood

RBC quality was assessed according to Brazilian legislation [16] and the manual for quality control of whole blood and components [21]. Samples were tested for changes in total hemoglobin, hematocrit, free hemoglobin, and degree of hemolysis. Hemoglobin (g/dL) and hematocrit (%) analyses were performed in an automated hematology counter. Free hemoglobin concentration (mg/mL) was measured in 20 μL supernatant after centrifugation at 2,500 rpm for 12 min, by the azide-methemoglobin method, in a spectrophotometer (HemoCue^®^ plasma low, Sweden) read at 570 and 880 nm [21]. The degree of hemolysis degree was expressed as percentage hemolysis, calculated as (100 –hematocrit) × free hemoglobin/total hemoglobin.

### Carboxyhemoglobin levels

COHb levels were determined in 5-mL samples by the co-oximetry method [22], at the HCPA Clinical Pathology Laboratory. Results were expressed as percentage of CO-saturated blood.

### Statistical analysis

Results were entered into a database and tested for normality of distribution by the Shapiro-Wilk method. Parametric data were analyzed by Student’s T-test for between-group comparisons. Nonparametric data were analyzed by the Kruskal–Wallis test followed by Dunn’s test. The chi-square test was used to compare categorical variables between groups. Generalized estimating equations (GEE) were used for between-group comparisons at the different time points of analysis (0, 15, and 30 days) [23]. Spearman correlation coefficients were calculated to determine the associations of abstinence interval and number of cigarettes smoked per day with COHb levels. All analyses were performed using the Statistical Package for Social Sciences (SPSS), Version 21.0. Data are presented as mean ± standard error (SEM) or median (interquartile range) as appropriate. The significance level was set at P<0.05.

## Results

From October 2013 to September 2014, 14,428 donations were received by the HCPA Blood Bank, from a monthly average of 1,110 blood donors. Overall, 857 donations were provided by smokers, representing 5.9% of total donations. General characteristics of this smoking donor population are described in Table 1. More than 60% of RBC packs were from men, with a mean age of 38 ± 11 years. Free hemoglobin and systolic and diastolic blood pressures were slightly higher in men than in women, while heart rate was slightly lower in men. Both men and women were generally overweight (BMI > 25 kg/m^2^), and depression and hypertension were the diseases most frequently reported during the mandatory interview for pre-donation screening. Alcohol drinking was reported by 17% of smokers. Furthermore, 3.7% of men and 2.9% of women who smoked tobacco also reported use of illicit drugs, such as cocaine and marijuana. Of 1,110 smoker blood donors, only four (all male) reported any discomfort after donation, such as dizziness and tingling.

**Table 1:** Characteristics of smoker blood donors enrolled from October 2013 to September 2014 at the HCPA Blood Bank, Porto Alegre, Brazil.

Variable	Female (n = 311)	Male (n = 546)	*P*
Frequency (%)	36.3	63.7	**<0.001**
Age (years)	37.5 ± 11.0	38.8 ± 11,5	0.116
Hemoglobin (g/dL)	14.0 ± 1.0	15.3 ± 1.2	**<0.001**
Systolic blood pressure (mmHg)	124.4 ± 17.0	129.7 ± 16.4	**<0.001**
Diastolic blood pressure (mmHg)	77.0 ±11.0	79.7 ± 11.0	**<0.001**
Heart rate (bpm)	80.3 ± 10.5	76.9 ± 11.4	**<0.001**
Body mass index (kg/m^2^)	26.9 ± 5.0	27.0 ± 4.4	0.775
Comorbidities that did not preclude donation, % (n)	11.6 (36)	8.1 (44)	0.116
Alcohol use, % (n)	17.2 (56)	17.0 (94)	1.000
Illicit drug use, % (n)	2.9 (9)	3.7 (20)	0.687
Adverse reactions, % (n)	0 (0)	0.7 (4)	0.303

Table 2 shows the demographic characteristics of age- and gender-matched smoker and control (non-smoker) blood donors. In this sample, the majority of RBC packs were also from men (~70%). The mean donor age was 32.3 ± 4.7 years, and most lived in Porto Alegre. A higher level of education was more frequent in non-smokers. In the smoker group, the median tobacco burden in pack-years was 14.0 [10.0 to 22.5]. History of smoking showed a non-significant difference between women and men, with a median of 10.5 [6.4 to 22.4] and 15.0 [10.0 to 22.5] pack-years, respectively (P = 0.390). Three donors (two male and one female) had a smoking history of more than 37 pack-years.

**Table 2:** Demographic characteristics of smoking and non-smoking blood donors from the HCPA Blood Bank included in the cohort study from June to September 2014.

	Non-smokers (n = 31)	Smokers (n = 31)
Men (%)	67.7 (21)	67.7 (21)
Age (years)	32.2 ± 4.7	32.4 ± 4.8
Marital status		
Single (%)	42 (13)	51.6 (16)
Married, divorced or widowed (%)	58 (18)	48.4 (15)
Educational attainment^a^ (%)		
Primary	6.4 (2)	12.9 (4)
Secondary	48.4 (15)	64.5 (20)
Higher	45.2 (16)	22.6 (7)
Place of residence (%)		
Porto Alegre	70 (24)	61 (19)
Other cities	30 (7)	39 (12)
Smoking history (pack-years)	-	14 [10–22.5]

^a^Considering both completed and not completed.

Data presented as mean ± SD for continuous variables and n (%) for categorical variables. Pack-years calculated by multiplying the number of cigarette packs smoked per day by the number of years of smoking.

**Fig 1:**
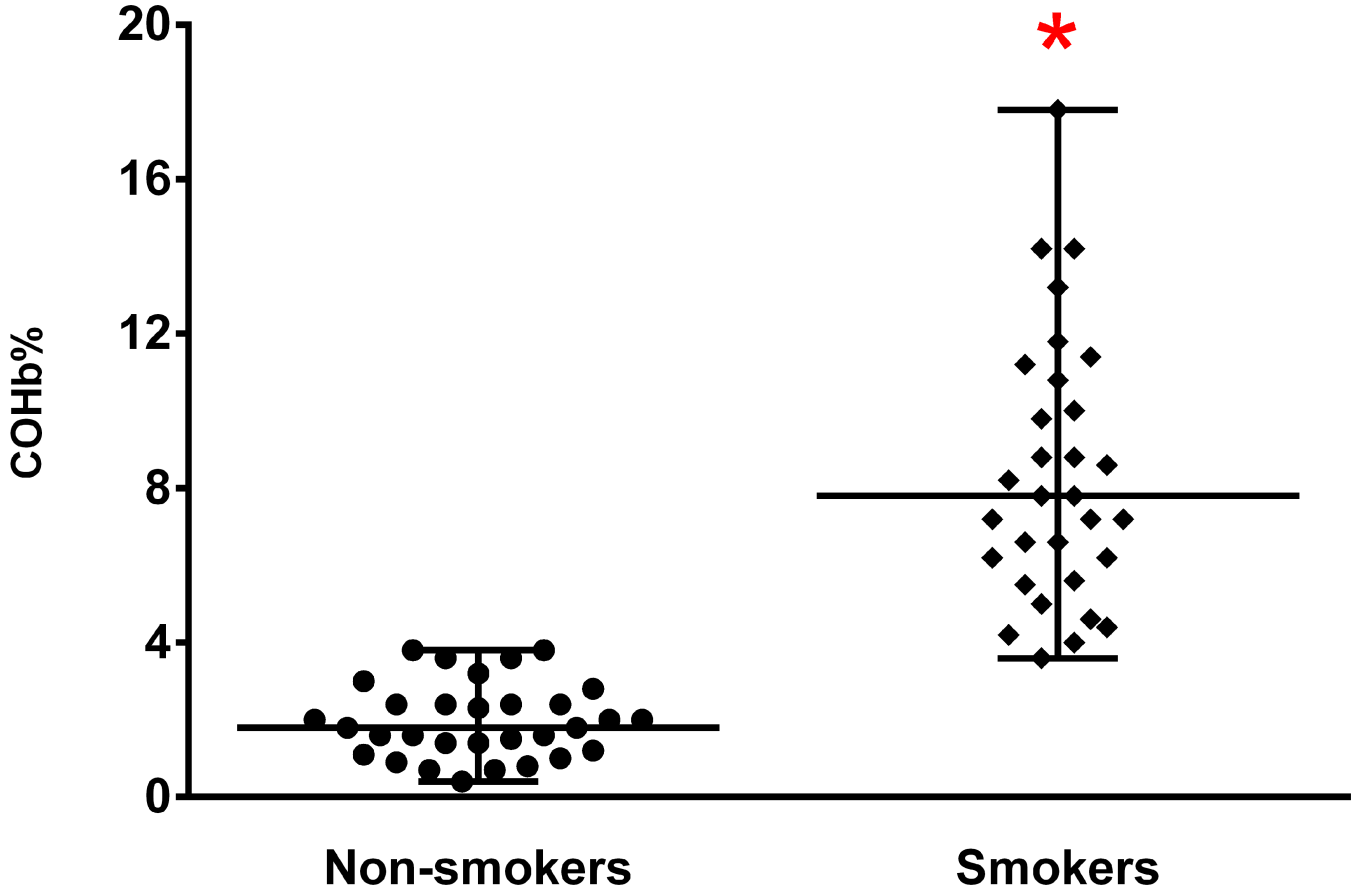
Carboxyhemoglobin (COHb) levels on the day of donation in packed red blood cells from smoker and non-smoker donors, HCPA Blood Bank, Brazil. N = 31/group; Kruskal-Wallis + Dunn’s test; P < 0.001.

Concerning blood quality, we found that donor smoking did not affect parameters such as hematocrit, hemoglobin, or hemolysis (S1 Table). These blood parameters changed slightly over time with storage, without, however, losing the properties required for appropriate and safe transfusion according to national standards [16]. COHb levels in RBC packs from smokers were fourfold higher than in packs from non-smokers (P < 0.001 S1 Table, Fig 1). Over storage time, COHb levels fell approximately 10% (P < 0.001) in the RBC packs donated from smokers, but remained four times higher than in RBC packs from non-smokers (S1 Table). In RBC packs from smokers, COHb levels ranged from 3.6 to 17.8% at time zero, and 14 RBC packs (45% of samples) presented values higher than 8%. There was an interaction between smoking status and COHb levels in the RBC packs (S1 Table).

**Fig 2:**
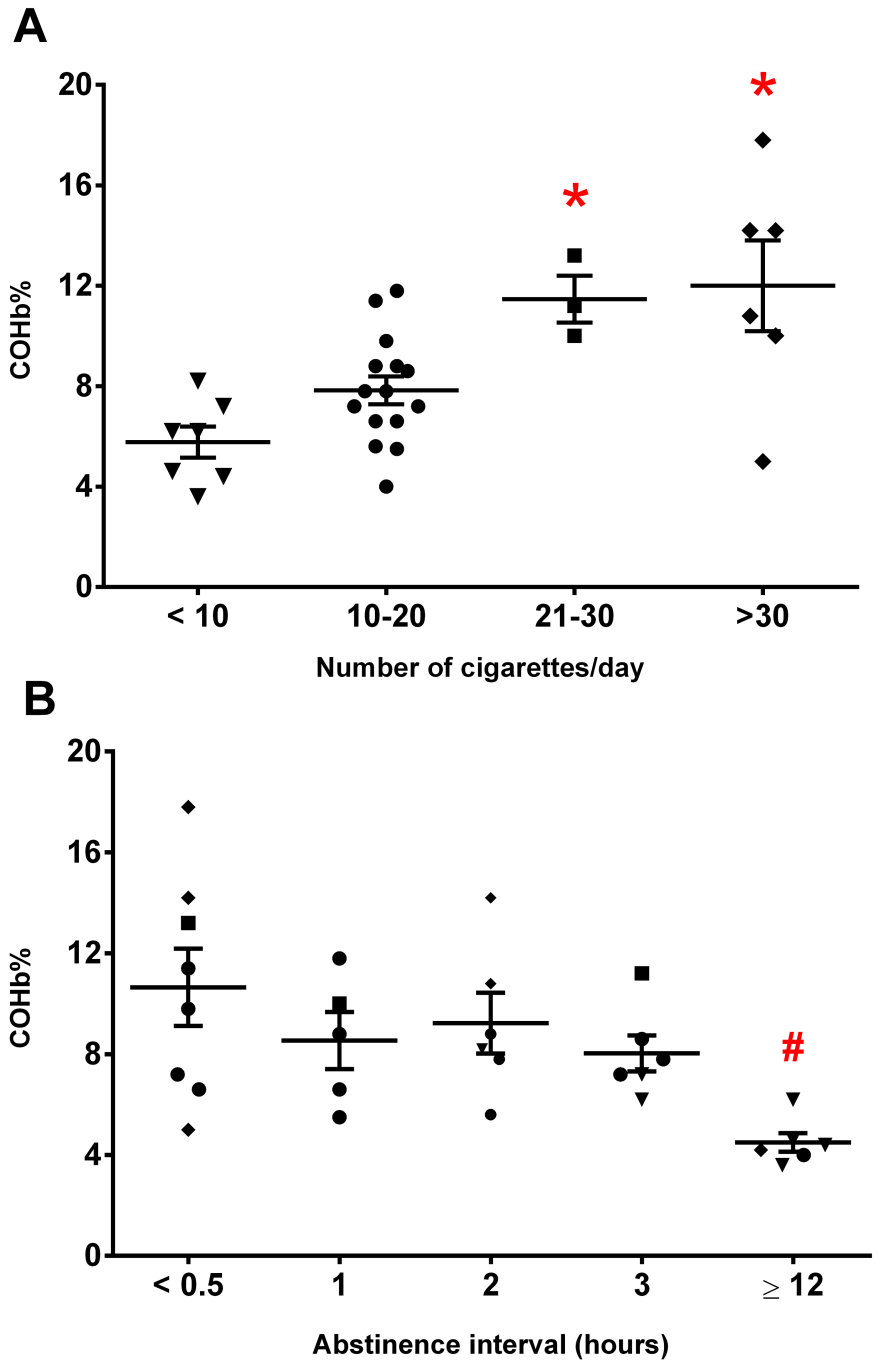
Carboxyhemoglobin (COHb) levels on the day of donation in packed red blood cells from smoker donors according to (A) number of cigarettes smoked per day (* different from < 10 and 10–20 cigarettes/day; P = 0.045) and (B) abstinence interval (▼ < 10; ● 10–20; ■ 21–30; and ♦ > 30 cigarettes/day; # different from ≤3 hours of abstinence; P = 0.008). N = 31/group; Kruskal-Wallis + Dunn’s test.

Spearman’s test revealed a direct correlation between COHb levels and the number of cigarettes smoked per day (r = 0.51; P = 0.003; Fig 2A). The median COHb levels from subjects who smoked fewer than 10 cigarettes per day was 6.2% [4.4%-7.2%]. In donors who smoked 20 cigarettes or more per day, values were twice as high (P = 0.045; Fig 2A). Additionally, we found a significant inverse correlation between COHb levels and abstinence time (r = -0.57; P < 0.001; Fig 2B). COHb levels decreased around 60% in the RBC packs donated by smokers who had remained abstinent of cigarettes for 12h or more before donation (P = 0.0169; Fig 2B). There were no differences in hemolysis between packs from smokers and non-smokers, regardless of number of cigarettes smoked per day or abstinence time since last cigarette (S1 Table).

## Discussion

Despite an alarming rise in the prevalence of tobacco use worldwide, smoking habits are not carefully investigated prior to blood donation [15,16]. In this study, we found that 5.9% of donors to the HCPA Blood Bank were smokers. We also found that, although smoking habit did not affect the overall quality of RBCs significantly, it did increase average COHb levels in RBC packs by up to four times, in a manner dependent to overall smoking burden and time elapsed since the last cigarette smoked before donation.

The prevalence of smoker donors found in this sample (5.9%) was lower than in the general population of Porto Alegre (14.2%). Smokers were also less represented among blood donors than in the general population in Lages, another city in Southern Brazil (12% vs. 20%, respectively); in the Netherlands (17.1% vs. 31.8%, respectively); and in Denmark (11.8% vs. 17.6%, respectively) [24,25]. Because smoking predicts comorbidity [26] and loss of eligibility, we suggest that smokers are less likely to donate, contributing to the lower prevalence of smoking among blood donors.

In this study, replicating prior results from the literature, we showed that smoking was more prevalent in men than in women, and that blood pressure and hemoglobin levels were slightly higher in male than in female smokers [9,27,28]. As in previous studies, we did not find changes in parameters commonly used to assess quality of packed RBCs for transfusion [16,28–30]. An in vitro study has shown that exposure of isolated erythrocytes to cigarette smoke increases hemolysis and COHb levels, with a correlation to increasing oxidative stress parameters [31]. Moreover, chronic cigarette smoking alters the rheological behavior of RBCs, decreasing their fluidity and compromising the flow properties of blood [32,33]. Further studies are needed to investigate changes in inflammatory parameters or the presence of heavy metals in RBC packs from smoker donors and their potential implications for the recipient [19]. One study found that active and passive tobacco smokers show higher blood lead levels than never-smokers [34].

Blood COHb levels in non-smokers range from 0.3% to 0.7% in rural areas and 1% to 2% in urban areas, rising to 3% and 8% respectively in smokers [1,9,17]. We found average COHb levels of 2% in RBC packs from non-smokers and 8% in those from smokers. Blood samples obtained from non-smoking taxi drivers in Porto Alegre showed COHb levels around 2%; this relatively high level was attributed to environmental pollution [35]. Impressively, 45% of packs from smoker donors showed COHb levels higher than 8%, in a manner dependent of the number of cigarettes smoked per day and time elapsed since the last cigarette smoked before donation. As expected, COHb levels were higher in RBC packs from donors who smoked more than 20 cigarettes daily and from those who had been abstinent for shorter than 12h. Thus, according to our results, the daily cigarette habit and the length of abstinence from smoking before donation may improve the quality and safety of donated blood.

Blood transfusion provides great clinical benefit. Thus, RBC packs donated by smokers should not be rejected, but may be subject to additional monitoring to avoid potential hazards to the health of the recipient. Although we cannot draw conclusive evidence from our results, additional studies conducted in vitro or in animal models should help establish safe limits for COHb in RBCs. Until there, we suggest that simple, low-cost approaches be adopted by blood banks to minimize risks, such as including a question about tobacco smoking habits in pre-donation screening questionnaires. In future, once a safe reference range for COHb levels from donated RBC packs has been established, such levels can be monitored immediately after extraction of blood components, helping prevent transfusion of COHb-rich blood to high-risk patients such as cyanotic neonates or older adults with heart disease [36–38]. Indeed, the magnitude of the effect of COHb levels in the recipient of packed RBCs donated by a smoker depends on factors such as the volume to be transfused and the recipient’s weight and previous health status [17,18,36,37]. In an adult recipient, transfusion of a single RBC pack (~250 mL) may not represent a risk even if COHb levels are elevated, as these will be diluted in the total blood circulation. However, when larger volumes of blood need to be transfused, such as during major surgery or in newborns (which often weigh less than 2.5 kg), COHb levels in RBC packs may be relevant [27,36,37]. One case study showed that, during congenital heart surgery in a newborn, COHb levels in blood reached 3.7% after infusion of a single RBC pack from a smoker donor containing 7.2% COHb; the patient’s gas-exchange capacity was reduced to dangerously low levels [27]. In adults with coronary artery disease, COHb levels higher than 2% can aggravate symptoms of angina, prolonging chest pain and decreasing exercise capacity [38]. These studies indicate the relevance of COHb levels for safe transfusion.

Distribution of COHb follows a two-compartment model, with a half-life of 1.6h for the first compartment and 30.9h for the second [39]. According to WHO, “recreational” drug use before donation is accepted if no signs of intoxication are present, as there is no clinical evidence that recent use by a donor causes harm to the recipient [40]. Guidelines for blood transfusion services in Brazil and in other countries do not establish rules for smoker donors, except that they should remain abstinent for 2h before and after donation to avoid discomfort [15,16,41]. Some authors have suggested cigarette abstinence for at least 24h before blood donation [18]. However, such a restrictive rule could decrease blood donation from tobacco-dependent individuals. In our sample, we found that abstaining from smoking for 12h was enough to reduce COHb levels. Implementing a restriction rule based on this parameter would be reasonable, since heavy smokers will not remain abstinent for longer than 12h.

In summary, we found that, although smoking did not compromise the quality of red blood cells as assessed by conventional quality control tests, it increased COHb levels up to four times. Abstinence from smoking for 12h or more and smoking fewer than 20 cigarettes per day helped to decrease these levels. Although clinical trials are needed to establish the extent of the hazard caused by transfusion of RBC packs donated by smokers, the potential harm of these packs cannot be ignored, especially in neonates and other high-risk recipients. Given the increasing prevalence of tobacco use worldwide, we suggest blood banks recommend 12h of tobacco abstinence before donation and analyze COHb levels in donated RBCs to increasing the safety of transfusion. We hope the results of this study will inspire future research to explore additional toxicological parameters that may help to improve transfusion safety.

## Supporting information

S1 Table Quality of packed red blood cells among smoking and nonsmoking donors in the Hospital de Clínicas de Porto Alegre, RS. (DOCX)

## References

[1] WHO. World Health Organization. Raisin tax on tobacco—What you need to know [Internet]. 9 Nov 2016 [cited 8 Jul 2017]. http://apps.who.int/iris/bitstream/10665/112841/1/WHO_NMH_PND_14.2_eng.pdf

[2] Geiss O, Kotzias D. Tobacco, Cigarettes and Cigarette Smoke—An Overview [Internet]. 2007. http://publications.jrc.ec.europa.eu/repository/handle/111111111/8885

[3] Eatough DJ, Benner CL, Bayona JM, Richards G, Lamb JD, Lee ML, et al Chemical composition of environmental tobacco smoke. 1. Gas-phase acids and bases. Environ Sci Technol. 1989;23: 679–687. 10.1021/es00064a006

[4] Talhout R, Schulz T, Florek E, van Benthem J, Wester P, Opperhuizen A. Hazardous Compounds in Tobacco Smoke. Int J Environ Res Public Health. 2011;8: 613–628. 10.3390/ijerph8020613 21556207 PMC3084482

[5] Milnerowicz H, Sciskalska M, Dul M. Pro-inflammatory effects of metals in persons and animals exposed to tobacco smoke. J Trace Elem Med Biol Organ Soc Miner Trace Elem GMS. 2015;29C: 1–10. 10.1016/j.jtemb.2014.04.008 24916792

[6] Goldstein M. Carbon monoxide poisoning. J Emerg Nurs JEN Off Publ Emerg Dep Nurses Assoc. 2008;34: 538–542. 10.1016/j.jen.2007.11.014 19022078

[7] Aronow WS, O’Donohue WJ, Freygang J, Sketch MH. Carboxyhemoglobin levels in banked blood. Chest. 1984;85: 694–695. 6713980 10.1378/chest.85.5.694

[8] Freeman R, Perks D. Incidence and range of carboxyhaemoglobin in blood for transfusion. Anaesthesia. 1990;45: 581–583. 2386284 10.1111/j.1365-2044.1990.tb14836.x

[9] Madany IM. Carboxyhemoglobin levels in blood donors in Bahrain. Sci Total Environ. 1992;116: 53–58. 1411494 10.1016/0048-9697(92)90364-x

[10] Bolton-Maggs PHB, Cohen H. Serious Hazards of Transfusion (SHOT) haemovigilance and progress is improving transfusion safety. Br J Haematol. 2013;163: 303–314. 10.1111/bjh.12547 24032719 PMC3935404

[11] Brand A. Immunological complications of blood transfusions. Presse Medicale Paris Fr 1983. 2016;45: e313–324. 10.1016/j.lpm.2016.06.024 27499223

[12] Garraud O, Filho LA, Laperche S, Tayou-Tagny C, Pozzetto B. The infectious risks in blood transfusion as of today—A no black and white situation. Presse Medicale Paris Fr 1983. 2016;45: e303–311. 10.1016/j.lpm.2016.06.022 27476017

[13] Strobel E. Hemolytic Transfusion Reactions. Transfus Med Hemotherapy Off Organ Dtsch Ges Transfusionsmedizin Immunhamatologie. 2008;35: 346–353. 10.1159/000154811 21512623 PMC3076326

[14] El Kenz H, Van der Linden P. Transfusion-related acute lung injury. Eur J Anaesthesiol. 2014;31: 345–350. 10.1097/EJA.0000000000000015 24892308

[15] Fung MK, Grossman, Brenda J, Hillyer C, Westhoff CM. American Association of Blood Banks: Advancing Transfusion and Cellular Therapies Worldwide. 18th ed. Bethesda, MD: AABB; 2014.

[16] BRASIL. Ministério da Saúde. Portaria 158. Redefine o regulamento técnico de procedimentos hemoterápicos. 2016;DOU, 05/02/2016: 25(1):37.

[17] Aberg A-M, Sojka BN, Winsö O, Abrahamsson P, Johansson G, Larsson JE. Carbon monoxide concentration in donated blood: relation to cigarette smoking and other sources. Transfusion (Paris). 2009;49: 347–353. 10.1111/j.1537-2995.2008.01951.x 18980621

[18] Symvoulakis EK, Vardavas CI, Fountouli P, Stavroulaki A, Antoniou KM, Duijker G, et al Time interval from cigarette smoke exposure to blood donation and markers of inflammation: should a smoking cut-off be designated? Xenobiotica Fate Foreign Compd Biol Syst. 2010;40: 613–620. 10.3109/00498254.2010.500745 20602565

[19] Delage G, Gingras S, Rhainds M. A population-based study on blood lead levels in blood donors. Transfusion (Paris). 2015;55: 2633–2640. 10.1111/trf.13199 26172273

[20] Abramson JH. WINPEPI updated: computer programs for epidemiologists, and their teaching potential. Epidemiol Perspect Innov EPI. 2011;8: 1 10.1186/1742-5573-8-1 21288353 PMC3041648

[21] Sakuma A, Ottoboni MAP, Sierra PC. Manual para controle da qualidade do sangue total e hemocomponentes. SIBRATEC São Paulo; 2011.

[22] Beutler E, West C. Simplified determination of carboxyhemoglobin. Clin Chem. 1984;30: 871–874. 6723043

[23] Guimarães LSP, Hirakata VN. Uso do Modelo de Equações de Estimativas Generalizadas na análise de dados longitudinais. Clin Biomed Res. 2013;32: 503–511.

[24] Atsma F, Veldhuizen I, de Vegt F, Doggen C, de Kort W. Cardiovascular and demographic characteristics in whole blood and plasma donors: results from the Donor InSight study. Transfusion (Paris). 2011;51: 412–420. 10.1111/j.1537-2995.2010.02867.x 20804526

[25] Spada C, Treitinger A, Souza MA. Smoking prevalence in donors from the mountain region of Santa Catarina—Brazil. Rev Bras Hematol E Hemoter. 2006;28: 19–23. 10.1590/S1516-84842006000100006

[26] Hughes JR. Comorbidity and smoking. Nicotine Tob Res Off J Soc Res Nicotine Tob. 1999;1 Suppl 2: S149–152; discussion S165-166. 10.1080/14622299050011981 11768173

[27] Ehlers M, Labaze G, Hanakova M, McCloskey D, Wilner G. Alarming levels of carboxyhemoglobin in banked blood. J Cardiothorac Vasc Anesth. 2009;23: 336–338. 10.1053/j.jvca.2008.12.006 19201206

[28] Hampson NB. Stability of carboxyhemoglobin in stored and mailed blood samples. Am J Emerg Med. 2008;26: 191–195. 10.1016/j.ajem.2007.04.028 18272101

[29] Aubron C, Bailey M, McQuilten Z, Pilcher D, Hegarty C, Martinelli A, et al Duration of red blood cells storage and outcome in critically ill patients. J Crit Care. 2014;29: 476.e1–8. 10.1016/j.jcrc.2014.01.006 24559574

[30] Koch CG, Figueroa PI, Li L, Sabik JF, Mihaljevic T, Blackstone EH. Red blood cell storage: how long is too long? Ann Thorac Surg. 2013;96: 1894–1899. 10.1016/j.athoracsur.2013.05.116 24090578

[31] Gangopadhyay S, Vijayan VK, Bansal SK. Lipids of erythrocyte membranes of COPD patients: a quantitative and qualitative study. COPD. 2012;9: 322–331. 10.3109/15412555.2012.668581 22497562

[32] Ernst E. Haemorheological consequences of chronic cigarette smoking. J Cardiovasc Risk. 1995;2: 435–439. 8749271 10.1177/174182679500200508

[33] Pretorius E. Transfusion medicine illustrated. Smokers as blood donors: what do their red blood cell membranes tell us? Transfusion (Paris). 2014;54: 266 10.1111/trf.12338 24517130

[34] Mannino DM, Homa DM, Matte T, Hernandez-Avila M. Active and passive smoking and blood lead levels in U.S. adults: data from the Third National Health and Nutrition Examination Survey. Nicotine Tob Res Off J Soc Res Nicotine Tob. 2005;7: 557–564. 10.1080/14622200500185264 16085527

[35] Brucker N, Moro AM, Charão MF, Durgante J, Freitas F, Baierle M, et al Biomarkers of occupational exposure to air pollution, inflammation and oxidative damage in taxi drivers. Sci Total Environ. 2013;463–464: 884–893. 10.1016/j.scitotenv.2013.06.098 23872245

[36] Collard KJ. Transfusion related morbidity in premature babies: Possible mechanisms and implications for practice. World J Clin Pediatr. 2014;3: 19–29. 10.5409/wjcp.v3.i3.19 25254181 PMC4162441

[37] Valieva OA, Strandjord TP, Mayock DE, Juul SE. Effects of Transfusions in Extremely Low Birth Weight Infants: A Retrospective Study. J Pediatr. 2009;155: 331–37.e1. 10.1016/j.jpeds.2009.02.026 19732577 PMC3038786

[38] Aronow WS. Aggravation of angina pectoris by two percent carboxyhemoglobin. Am Heart J. 1981;101: 154–157. 7468415 10.1016/0002-8703(81)90658-x

[39] Cronenberger C, Mould DR, Roethig H-J, Sarkar M. Population pharmacokinetic analysis of carboxyhaemoglobin concentrations in adult cigarette smokers. Br J Clin Pharmacol. 2008;65: 30–39. 10.1111/j.1365-2125.2007.02974.x 17764477 PMC2291280

[40] WHO. Blood donor selection. In: WHO [Internet]. 2012 [cited 9 Jul 2017]. http://www.who.int/bloodsafety/publications/bts_guideline_donor_suitability/en/

[41] UKBTS. Guidelines for the blood transfusion services in the United Kingdom. 8th ed Kingdom: TSO; 2013.

