# Supplementary material for: Smoking fewer than 20 cigarettes per day and remaining abstinent for more than 12 hours reduces carboxyhemoglobin levels in packed red blood cells for transfusion
Source: PLoS One. 2018 Sep 26;13(9):e0204102. doi: 10.1371/journal.pone.0204102 (PMC6157890; doi:10.1371/journal.pone.0204102)
Supplement: S1 Table — (DOCX) [file pone.0204102.s001.docx]

**Table S1. Quality of packed red blood cells among smoking and nonsmoking donors in the Hospital de Clínicas de Porto Alegre, RS.**

| **Variable** | **Time**  **following**  (days) | **Group** | | | **P**  **_Non-smokers × smokers_** | **P**  **_0 × 15 × 30 days_** | | **P**  **_Interaction_** |
| --- | --- | --- | --- | --- | --- | --- | --- | --- |
|  |  | **Non-smokers**  (n = 31) | **Smokers**  (n = 31) | |  |  |  |  |
| Hematocrit (%) | 0 | 73.2 ± 0.6 | 73.44 ± 0.54 | 0.690 | | **<0.001** | 0.678 | |
|  | 15 | 75.6 ± 0.6^b^ | 76.42 ± 0.77^b^ |  |  |  |  |  |
|  | 30 | 74.6 ± 0.6 | 74.55 ± 0.75 |  |  |  |  |  |
| Hemoglobin (g/dL) | 0 | 23.3 ± 0.2 | 23.58 ± 0.19 | 0.651 | | **<0.001** | 0.391 | |
|  | 15 | 23.7 ± 0.2^b^ | 23.88 ± 0.22^b^ |  |  |  |  |  |
|  | 30 | 23.5 ± 0.2 | 23.49 ± 0.22 |  |  |  |  |  |
| Total Hemoglobin (g/pc) | 0 | 66.3 ± 1.3 | 67.58 ±1.16 | 0.584 | | **<0.001** | 0.359 | |
|  | 15 | 67.4 ± 1.4^b^ | 68.49 ±1.31^b^ |  |  |  |  |  |
|  | 30 | 66.8 ± 1.4 | 67.32 ±1.23 |  |  |  |  |  |
| Free Hemoglobin (g/dL) | 0 | 0.018 ± 0.004 | 0.026 ± 0.007 | 0.268 | | **<0.001** | 0.517 | |
|  | 15 | 0.130 ± 0.170^b^ | 0.170 ± 0.350^b^ |  |  |  |  |  |
|  | 30 | 0.230 ± 0.240^c^ | 0.290 ± 0.460^c^ |  |  |  |  |  |
| Hemolysis degree (%) | 0 | 0.019 ± 0.004 | 0.026 ± 0.006 | 0.316 | | **<0.001** | 0.693 | |
|  | 15 | 0.128 ± 0.013^b^ | 0.148 ± 0.019^b^ |  |  |  |  |  |
|  | 30 | 0.247 ± 0.023^c^ | 0.288 ± 0.039^c^ |  |  |  |  |  |
| Carboxyhemoglobin (%) | 0 | 2.0 ± 0.2 | 8.3 ± 0.6^a^ |  | |  |  | |
|  | 15 | 1.6 ± 0.2 | 7.3 ± 0.6^a,b^ | **<0.001** | | **<0.001** | **0.040** | |
|  | 30 | 1.7 ± 0.1 | 7.6 ± 0.5^a,c^ |  | |  |  | |

Results showed no differences between smokers and nonsmokers except for carboxyhemoglobin levels. Blood components significantly changed along the storage time (0 × 15 × 30 days) however the magnitude of the effect did not surpass limits considered normal, except for carboxyhemoglobin that not present limits of normality established yet. Results presented as mean ± S.E.M. Significant *P* highlighted in bold (GEE + Bonferroni test); a: different from nonsmokers; b: different from day 0; c: different from day 0 and 15. Excepts for carboxihemoglobina that levels
